# Are We Longer Lived Than Our Fathers?

**Published:** 1897-06

**Authors:** 


### Are We Longer-Lived than Our Fathers?

This question is answered in the affirmative by *The Lancet,* and it gives credit for the fact to the medical profession. It says:

"The mean duration of life among males has increased under the influence of preventive medicine by four years during the past fifty years. The increase of longevity among females has been . . . five years in the same period. Some are inclined to depreciate the value of this increase of the mean duration of life in England in recent years, because it is mainly due to the reduction of mortality in childhood. It is indeed true that after middle life the expectation of life has not increased, and has, indeed, slightly declined. The effect of this is, however, far more than counterbalanced by the largely increased proportion of persons who survive childhood and reach middle life. Thus, as has been pointed out, there has been a very considerable increase in the proportional number of years lived by succeeding generations between the most useful age-period of from twenty to sixty years. This may clearly be claimed as one of the results of the labors of preventive medicine. It remains to be seen whether further sanitary progress will make it possible to entirely counteract the effect of the increasing intensity of competition and the struggle for existence and survival which undoubtedly are now depressing the expectation of life after middle age. If, therefore, longevity signifies mean duration of life, there can be no doubt about its increase in England in recent years. If, however, by longevity is meant the chance of middle-aged persons reaching extreme old age, English life tables do not supply so satisfactory an answer to our question."

